# Adynamic response to cold pain reflects dysautonomia in type 1 diabetes and polyneuropathy

**DOI:** 10.1038/s41598-023-37617-9

**Published:** 2023-07-13

**Authors:** Thomas Arendt Nielsen, Søren Lundbye-Christensen, Yoanna Krasimirova Dimitrova, Sam Riahi, Birgitte Brock, Asbjørn Mohr Drewes, Christina Brock

**Affiliations:** 1 Department of Clinical Medicine, Aalborg University, Aalborg, Denmark; 2 Department of Ophthalmology, Aalborg University Hospital, Aalborg, Denmark; 3 Department of Gastroenterology and Hepatology, Mech-Sense, Aalborg University Hospital, Aalborg, Denmark; 4 Unit of Clinical Biostatistics, Aalborg University Hospital, Aalborg, Denmark; 5 Department of Cardiology, Aalborg University Hospital, Aalborg, Denmark; 6 Steno Diabetes Center Copenhagen, Region Hovedstaden, Gentofte, Denmark; 7 Steno Diabetes Center North Denmark, Aalborg, Denmark

**Keywords:** Diabetes, Cardiology, Neurodegeneration

## Abstract

Cardiac autonomic neuropathy (CAN), widely assessed by heart rate variability (HRV), is a common complication of long-term diabetes. We hypothesized that HRV dynamics during tonic cold pain in individuals with type 1 diabetes mellitus (T1DM) could potentially demask CAN. Forty-eight individuals with long-term T1DM and distal symmetrical polyneuropathy and 21 healthy controls were included. HRV measures were retrieved from 24-h electrocardiograms. Moreover, ultra-short-term HRV recordings were used to assess the dynamic response to the immersion of the hand into 2 °C cold water for 120 s. Compared to healthy, the T1DM group had expectedly lower 24-h HRV measures for most components (p < 0.01), indicating dysautonomia. In the T1DM group, exposure to cold pain caused diminished sympathetic (p < 0.001) and adynamic parasympathetic (p < 0.01) HRV responses. Furthermore, compared to healthy, cold pain exposure caused lower parasympathetic (RMSSD: 4% vs. 20%; p = 0.002) and sympathetic responses (LF: 11% vs. 73%; p = 0.044) in the T1MD group. QRISK3-scores are negatively correlated with HRV measures in 24-h and ultra-short-term recordings. In T1DM, an attenuated sympathovagal response was shown as convincingly adynamic parasympathetic responses and diminished sympathetic adaptability, causing chronometric heart rhythm and rigid neurocardiac regulation threatening homeostasis. The findings associate with an increased risk of cardiovascular disease, emphasizing clinical relevance.

## Introduction

Cardiac autonomic neuropathy (CAN) has a typical silent presentation, especially in the early stages. Thus it is a common under-recognized chronic complication and is an independent predictor and risk marker of cardiovascular morbidity and all-cause mortality^1–5^. CAN affect approximately 20% of unselected populations with type 1 and type 2 diabetes and up to 65% with increasing age and diabetes duration^6^. Recently HRV analyses have identified CAN in early disease stages in individuals with type 1 diabetes mellitus (T1DM)^7^.

Cardiovascular autonomic reflex tests are considered the gold standard for diagnosing CAN and include dynamic evaluation of short recordings of R-R intervals and blood pressure in response to postural change, deep breathing, handgrip, and the Valsalva maneuver^6,8^. However, heart rate variability (HRV), obtained from long- or short-term electrocardiograms, has been widely used in clinical research to describe neurocardiac regulation^2,8–10^. HRV indicates the variation in time between consecutive QRS complexes of normal sinus depolarizations (RR intervals). It measures the heart's adaptability, assessed as variations in the time (assessed in milliseconds) or frequency components (assessed as power). To obtain complementary information on central neurocardiac regulation, assessments of cardiac vagal tone (CVT) and cardiac sensitivity to the baroreflex (CSB) can be obtained, representing the efferent and afferent central parasympathetic neurocardiac regulation, respectively^11–13^.

Neurocardiac regulation secures the heart rhythm’s adaptability to sudden physical or psychological challenges, and several methods for sympathetic activation of the HRV response have been used, including pharmacological modulation via administering sympathomimetics, parasympatholytics, or physiological modulation, e.g., stress-induced acoustic startle and postural changes^14–19^. It has been reported that in healthy subjects^20–23^ and individuals with diabetes^20,24^, exposure to tonic cold pain increases plasma epinephrine and norepinephrine, indicative of a sympathetic response. This is further supported by findings in healthy controls, who showed increased heart rate, systolic and diastolic blood pressure, and an autonomic affective component (unpleasantness) of pain in response to cold pain^16–18,25–27^. However, high inter-individual variability has been reported, and conflicting reports exist^25^.

In individuals with diabetes, HRV measures are known to be reduced compared to healthy controls^11,27–31^, interpreted as a consequence of sympathetic dominance and/or parasympathetic withdrawal. The low-frequency (LF) component of HRV is thought to reflect the combined sympathetic and parasympathetic activity, whereas high-frequency (HF) is consistently reported as a proxy of parasympathetic activity. Thus, LF/HF ratio has been widely used to describe the sympathovagal balance. However, conflicting results exist, which may indicate both co-activation or co-inhibition of the two tonically activated branches, and thus, LF/HF may not always index autonomic balance accurately^7,10,32,33^. To the best of our knowledge, HRV and assessment of the dynamic frequency components and sympathovagal balance in response to and following tonic cold pain have not yet been studied in individuals with type 1 diabetes and polyneuropathy. Thus, it remains unclear whether HRV in such a provocative test can reveal robust dynamic differences as an indicator of dysfunctional neurocardiac regulation.

There is an unmet clinical need to develop provocative tests that could reveal early signs of CAN.

To establish the model, we suggest investigating the dynamic HRV response to exposure to tonic cold water in individuals with polyneuropathy. We hypothesized that exposure to tonic cold pain would lead to enhanced sympathetic drive in healthy in comparison to individuals with type 1 diabetes and polyneuropathy. Furthermore, we hypothesized that exposure to tonic pain would attenuate the dynamic sympathovagal balance response in participants with long-term type 1 diabetes and polyneuropathy compared to healthy controls. This study aimed to assess the dynamic HRV alterations of the sympathetic, parasympathetic, and sympathovagal balance of the neurocardiac regulation in response to tonic cold pain exposure in (1) healthy controls and (2) in individuals with T1DM and polyneuropathy. Moreover, we aimed to assess differences in classical 24-h HRV measures, baroreflex sensitivity, and centrally regulated CVT at rest between the two groups.

## Methods

This study was based on secondary analyses of baseline data from a larger randomized clinical trial (clinicaltrials.gov: NCT02138045)^34^.

### Study population

A total of 69 participants were recruited. The cohort consisted of 48 individuals (38 male and 10 female, median age 50 years (IQR 45–56) with long-term T1DM (median duration 31 years (IQR 25–40) and severe concomitant distal symmetric polyneuropathy (DSPN) diagnosed in accordance with the Toronto criteria^35^. Inclusion criteria were stable diabetes treatment at least three months before enrolment, 18–65 years of age, and body mass index (BMI) > 22. Exclusion criteria were type 2 diabetes, psychiatric disease, reduced kidney function (estimated glomerular filtration rate < 60 mL/min/1.37 m^2^), neurological disorders other than distal symmetric polyneuropathy, glycosylated hemoglobin (HbA1c) level < 7% (48 mmol/mol) and treatment for other endocrine disorders. The participants had no history of coronary disease, peripheral arterial disease, or cerebrovascular disease, except one participant who had previously had a cerebral sinus vein thrombosis**.** For comparison, the control group consisted of 21 age- and sex-matched healthy controls (15 male and six female, median age of 52 years (IQR 48–55) recruited for a randomized controlled trial (N-20090008) by our research group. Exclusion criteria were diabetes, neurological or endocrine disorders, psychiatric disease, reduced eGFR (< 60 mL/min/1.37 m^2^), and a history of cardiovascular disease. The estimated clinical risk of having a cardiovascular event within the next 10 years was calculated using the QRISK3 risk calculator (qrisk.org).

### Clinical biochemistry

Blood samples, including glycosylated hemoglobin (HbA1c), high-density lipoprotein (HDL), low-density lipoprotein (LDL), and creatinine, were collected and analyzed by the Department of Clinical Biochemistry, Aalborg University Hospital. HbA1c was analyzed using capillary electrophoresis (Sebia Capillarys 3, Sebia Ltd. Camberley, UK). HDL and LDL were analyzed using Abbott Alinity (Abbott, Abbott Park, Illinois, US). eGFR was calculated using the CKD-EPI formula, allowing kidney function assessment. Moreover, urine samples were collected to assess urine albumin creatinine ratio (UACR), allowing for the assessment of the presence of microalbuminuria/macroalbuminuria. UACR between 30 and 299 mg/g creatinine is defined as microalbuminuria, whereas values above 300 mg/g creatinine are defined as macroalbuminuria. Urine albumin and creatinine were analyzed using Abbott Alinity (Abbott, Abbott Park, Illinois, US).

### Heart rate variability

Twenty-four-hour continuous electrocardiograms (Holter monitoring) were recorded using Lifecard CF (Del Mar Reynolds Medical, Spacelabs Healthcare Inc.), according to internationally recommended standards^8^. The initial recording period comprised a 10‐min epoch where participants were instructed to relax. This was followed by 2 × 15‐min periods of guided respiratory rate of 15 breaths/min in the supine position, followed by the same respiration rate in standing position. Orthostatic hypotension was defined as > 20 mmHg reduction in systolic blood pressure between supine vs. standing position. All recordings were visually inspected for artifacts/ectopic beats/atrial fibrillation etc., and HRV measures were derived using Pathfinder and HRV Tools (Del Mar Reynolds Medical, Spacelabs Healthcare Inc.). The following time-domain components were derived: Standard deviation of NN (normal-to-normal beats) intervals (SDNN), mean standard deviation of all NN intervals for each 5-min segment of a 24-h recording (SDNNI), standard deviation of all NN intervals for each 5-min segment of a 24-h recording (SDANN), root mean square of successive differences (RMSSD) in NN intervals, mean RR interval and heart rate. By utilizing fast Fourier transformation, the following frequency-domain components were derived: total power (≤ 0.4 Hz), very-low-frequency (VLF, 0.0033 to 0.04 Hz), low-frequency (LF, 0.04–0.15 Hz), high-frequency power (HF, 0.15–0.4 Hz) and low-frequency/high-frequency ratio (LF/HF). All HRV indices were adjusted for baseline HRV. Definitions used in this study are based on Shaffer et al.^10^.

### Cardiac vagal tone and cardiac sensitivity to the baroreflex

Additionally, validated measures of CVT and CSB were recorded using a designated electronic device (Neuroscope, Medifit Instruments, Enfield, Essex, UK), providing real-time measures of efferent and afferent central neurocardiac regulation, e.g., parasympathetic brainstem influence of the heart (Neuroscope, Medifit Instruments, Enfield, Essex, UK)^13,34,36^. Finally, blood pressure was continuously measured using a blood pressure monitor (Omron M4, Hoofddorp, Netherlands).

### Tonic cold pain

The participants were situated in a 60-degree supine position and instructed to breathe spontaneously. The participants immersed their left open hand into circulating cooled water (2.0 °C ± 0.3 °C) and maintained it for 120 s or earlier if intolerable pain was reached. Following tonic cold pain exposure, participants were asked to rate the pain and unpleasantness intensity on a modified rating scale, organized as an 11-point Likert scale, ranging from 0 to 10 (0 = no pain, 10 = worst imaginable pain).

To derive the dynamic changes in the neurocardiac regulation assessed as a sympathetic and parasympathetic response, HRV was recorded continuously, starting 4 min before (pre-testing), during (initial response), and up to 16 min after (sustained and recovery responses). Subsequently, these recordings were divided into ultra-short HRV recordings of 2-min epochs^8,37^. To characterize the dynamic physiological pattern, HRV was derived from each of these epochs. To improve the robustness of our pre-testing baseline, we used the mean of the two epochs representing pre-tests (four to two and two to zero min before immersion).

### Statistics

Data management was carried out using Epidata Software® (The Epidata Association, Odense, Denmark), and statistical analysis was performed using Stata® (StataCorp LLC v. 17.0, Texas, USA). Data were tested for normality using Shapiro–Wilk and by visual inspection of histograms and Q–Q plots. Baseline characteristics and HRV measures are presented as the median and interquartile range (IQR). Differences between T1DM and the control group were compared using unpaired t-tests. Calculations of standard error, confidence intervals, and p-values were calculated using bootstrapping with 1000 replications to accommodate for violations in normality and correlation structure. Categorical data are presented as numbers in each group and compared using Fisher’s exact test. Spearman’s rank correlation was used to test for associations between QRISK3 and HRV recordings, CVT and CSB. Group differences in HRV, in response to tonic cold pain, were calculated using repeated measures-ANOVA and subsequentially by linear comparison of the measures. Pre-test values used in tonic cold pain were calculated as the average between two consecutive 2-min pre-test measurements, recorded 4 and 2 min before exposure. The main outcome was analyzed using crude differences and subsequently adjusted for age and BMI (adjusted differences) using regression analysis with bootstrapping with 1000 replications and reported as mean with confidence interval. Relative changes reported as percentage in HRV measures in response to tonic cold pain was calculated as the percentual differences between the pre-test and the 2 to 4-min epoch recordings. A p-value of < 0.05 was considered significant.

### Ethics approval and consent to participate

All participants gave written informed consent before enrolment, and ethical approval was granted by The Scientific Ethics Committee, The North Denmark Region (N-20130077). The study was performed in accordance with the Declaration of Helsinki and the International Council for Harmonization’s guidelines for Good Clinical Practice.

## Results

Data of all 69 participants were assessed. Two participants in the T1DM group were excluded due to noisy HRV data (less than 80% of the recording met the quality criteria), and one participant was excluded due to repeated episodes of bradycardia. As for the tonic cold pain response, seven participants in both the T1DM group and healthy controls group had missing data (they withdrew their hand before 120 s), or HRV data was too noisy and were therefore excluded from the analysis.

For comparison, the two groups were matched on sex, age, weight, smoking status, and BMI, see Table 1. Participants with T1DM and polyneuropathy had significantly slower nerve conduction tests, higher heart rate (HR), and systolic blood pressure (SBP) in both rest and during a controlled breathing regime. 90% had orthostatic hypotension as an indicator of CAN. The presence of polyneuropathy did not influence the objective small fiber response to heat or subjective pain ratings to cold pain; however, the magnitude of unpleasantness was lower in the T1DM group. All participants in the T1DM group had diabetic retinopathy, and 31% had an elevated UACR ratio. eGFR and LDL cholesterol were lower in the T1DM group than in healthy controls.

**Table 1:** Baseline characteristics.

Baseline characteristics	T1DM	Controls	p-value
	*n* = 48	*n* = 21	
Demographics			
Sex (M/F)	38/10	15/6	0.542
Age (year)	50 (45–56)	52 (48–55)	0.424
Weight (kg)	87 (78–97)	84 (72–98)	0.607
Smoking (Y/N)	10/32	4/17	0.466
Body mass index (kg/m^2^)	28 (25–31)	26 (24–28)	0.202
Duration of T1DM (year)	31 (25–40)	–	
QRISK^®^3 (%)	19.5 (13–28)	–	
Clinical biochemistry			
HbA1c (mmol/mol)	64.5 (58–72)	33.5 (33–35)	**< 0.001**
HDL cholesterol (mmol/l)	1.5 (1–2)	1.5 (1–2)	0.962
LDL cholesterol (mmol/l)	2.5 (2–3)	3.4 (3–4)	**< 0.001**
eGFR (CKD‐epi)	86 (68–90)	90 (82–90)	**0.042**
UACR < 30 mg/g	15/48	1/21	**0.027**
Cardiac derived parameters			
Ortostatic hypotension	43/48	–	
Resting HR (bpm)	73 (67–78)	60 (58–65)	**< 0.001**
Resting SBP (mmHg)	151 (139–163)	126 (120–137)	**< 0.001**
Resting DBP (mmHg)	83 (76–88)	77 (72–82)	0.080
Controlled breathing HR (bpm)	71 (68–81)	64 (61–70)	**0.001**
Controlled breathing SBP (mmHg)	144 (130–156)	121 (115–128)	**< 0.001**
Controlled breathing DBP (mmHg)	83 (74–88)	78 (70–84)	0.206
Medications			
Anti-hyperlipidemic medication (Y/N)	25/48	–	
Anti-hypertensive medication (Y/N)	33/48	1/21	**< 0.001**
Microvascular complications			
Retinopati (PDR/NPDR)	15/33	–	
Distal symmetrical polyneuropathy			
Sural sensory NCV (m/s)	40 (40–43)	49 (45–53)	**< 0.001**
Median motor NCV (m/s)	50 (46–52)	55 (54–57)	**< 0.001**
Median sensory NCV (m/s)	42 (37–47)	54 (53–59)	**< 0.001**
Thermal pain tolerance threshold (°C)	49 (47–49)	50 (49–50)	**0.029**
Michigan neuropathy screening score	4 (3–5)	–	
Painful neuropathy	14/48	–	

	*n* = 47	*n* = 17	
Tonic cold pain exposure			
Pain (scale 0–10)	8 (7–9)	8 (6–9)	0.991
Unpleasantness (scale 0–10)	8 (6–9)	9 (7–10)	**0.035**

Values are reported as median (IQR) or as categorical values.

*M/F* male/female, *Y/N* yes/no, *HbA1c* glycated hemoglobin, *LDL* low-density lipoprotein, *HDL* high-density lipoprotein, *UACR* urine albumin creatinine ratio, *HR* heart rate, *bpm* beats per minute, *SBP* systolic blood pressure, *DBP* diastolic blood pressure, *NCV* nerve conduction velocity, *m/s* meters/second.

Significant values are in bold.

### HRV based on 24-h recordings

Based on the 24-h recordings, both time- and frequency domain measures were lower in the T1DM group than in the healthy controls, indicating diabetes-induced dysautonomia (see Table 2). In the T1DM group, significant negative correlations were found between QRISK3 score and the following 24-h HRV measures; total power (r(42) =  − 0.41, p = 0.006), VLF (r(42) =  − 0.41, p = 0.005), LF (r(42) =  − 0.43, p = 0.004) and HF (r(42) =  − 0.33, p = 0.03).

**Table 2:** Differences in HRV measures between T1DM and control group at rest (24-h), CVT, and CSB between T1DM and healthy controls, with and without adjustment for age and BMI.

Neurocardiac measures	Raw data			Crude differences		Adjusted differences*	
	T1DM	Controls	p-value	T1DM vs. controls	p value	T1DM vs. controls	p-value
	*n* = 45	*n* = 21		*n* = 66		*n* = 66	
HRV: time-domain							
Mean RR (ms)	771 (100)	878 (49)	**< 0.0001**	− 98.1 (− 53.4; − 142.7)	**0.000**	− 92.0 (− 46.8; − 137.2)	**0.000**
RMSSD (ms)	18 (11)	24 (8)	**0.013**	− 93.4 (37.9; − 224.6)	0.163	− 107.6 (41.9; − 257.2)	0.158
SDNN (ms)	111 (23)	141 (31)	0.154	− 12.4 (− 5.3; − 19.5)	**0.001**	− 12.1 (− 5.0; − 19.2)	**0.001**
SDNNi (ms)	40 (20)	54 (18)	**0.001**	− 22.8 (− 10.0; − 35.6)	**0.000**	− 21.1 (− 7.4; − 34.8)	**0.003**
SDANN (ms)	99 (23)	124 (27)	**< 0.0001**	− 5.1 (− 1.2; − 9.0)	**0.010**	− 4.8 (− 0.6; − 9.0)	**0.024**

	*n* = 44	*n* = 21		*n* = 65		*n* = 65	
HRV: frequency-domain							
Total power (ms^2^)	1551 (1412)	2600 (1266)	**< 0.0001**	− 1182.5 (− 671.2; − 1693.8)	**0.000**	− 1221.2 (− 705.4; − 1736.9)	**0.000**
VLF (ms^2^)	1082 (875)	1666 (1004)	**< 0.0001**	− 780.3 (− 437.6; − 1122.9)	**0.000**	− 799.8 (− 474.8; − 1124.7)	**0.000**
LF (ms^2^)	379 (396)	650 (310)	**< 0.0001**	− 339.4 (− 158.5; − 520.3)	**0.000**	− 356.1 (− 184; − 528.1)	**0.000**
HF (ms^2^)	88 (130)	177 (116)	**0.032**	− 63.9 (− 4.2; − 123.6)	**0.036**	− 66.7 (− 6.7; − 126.6)	**0.029**
LF/HF (ms^2^)	4.0 (2.5)	4.9 (3.1)	0.143	− 0.8 (0.3; − 1.9)	0.155	− 0.9 (0.3; − 2.1)	0.162

	*n* = 36	*n* = 21		*n* = 57		*n* = 57	
Other autonomic tests							
CVT (lvs)	2.4 (2.8)	4.5 (2.1)	0.059	− 3.6 (0.1; − 7.3)	0.060	− 3.6 (0.2; − 7.4)	0.066
CSB (ms/mmHg)	1.4 (1.8)	2.9 (1.7)	**0.017**	− 1.3 (− 0.3; − 2.3)	**0.013**	− 1.3 (− 0.1; − 2.4)	**0.029**

Healthy controls represent the standard, and negative values represent diminished activity in HRV measures in the T1DM group. Raw data values are reported as median (IQR). Values for crude and adjusted differences (*adjusted for age and BMI) are reported as mean (confidence interval) and performed using bootstrapping with 1000 replications.

*RMSSD* root mean square of successive differences in NN intervals, *SDNN* standard deviation of NN intervals, *SDNNI* mean of the standard deviation of all NN intervals for each 5-min segment of a 24-h recording, *SDANN* standard deviation of all NN intervals for each 5-min segment of a 24-h recording, *VLF* very-low-frequency, *LF* low-frequency, *HF* high-frequency, *LF/HF* low-frequency/high-frequency ratio, *CVT* cardiac vagal tone, *CSB* cardiac sensitivity to the baroreflex.

Significant p-values are reported in bold.

### Complementary neurocardiac measures

CSB was lower in the T1DM group than in healthy controls. A tendency towards decreased CVT in the T1DM group was found, but this did not meet significancy (p = 0.059).

### Dynamic HRV responses in the healthy controls group

Compared to RR-intervals in the pre-testing period, the mean RR decreased for all time points following tonic cold pain (p < 0.047). In contrast, SDNN (p = 0.015) and RMSSD (p < 0.001) increased in the initial response to tonic cold pain exposure. Furthermore, LF (p = 0.031) and HF (p = 0.038) increased immediately after cold pain exposure compared to the pre-testing period. No significant changes were found for total power, VLF, and LF/HF. A notable late sympathetic shoot was found for LF/HF in the sustained response to tonic cold pain exposure.

### Dynamic HRV responses in the T1DM group

When compared to RR-intervals in the pre-testing period, mean RR (p < 0.001) and RMSSD (p = 0.040) decreased as an initial response to tonic cold pain. Interestingly, this was accompanied by decreased LF (p = 0.004) and LF/HF ratio (p < 0.001). In the sustained response (6 to 8 min) (p = 0.015) and recovery phase (8 to 10 min) (p < 0.00), mean RR increased. Neurocardiac adaptability of the heart rate, reflected in the SDNN measure, was not evident until the recovery epochs at 10–12 min (p = 0.026) and 12–14 min (p = 0.005) after tonic cold pain. No significant changes were found for total power or HF.

Interestingly, QRISK3 scores were negatively correlated to alterations in SDNN (r(39) =  − 0.40, p = 0.009), total power (r(39) =  − 0.44, p = 0.004), VLF (r(39) =  − 0.50, p = 0.001) LF (r(39) =  − 0.46, p = 0.003), and HF (r(39) =  − 0.32, p = 0.042) component during tonic cold pain exposure. These correlations remained present 2 to 4 min and 4 to 6 min following exposure (p < 0.006). RMSSD showed a tendency towards a negative correlation with the QRISK3 score during exposure (p = 0.06) and a significant negative correlation to the QRISK3 score 2 to 4 min following exposure (r(39) =  − 0.4, p = 0.008).

### Differences in HRV responses to tonic cold pain between T1DM and healthy controls

**Figure 1:**
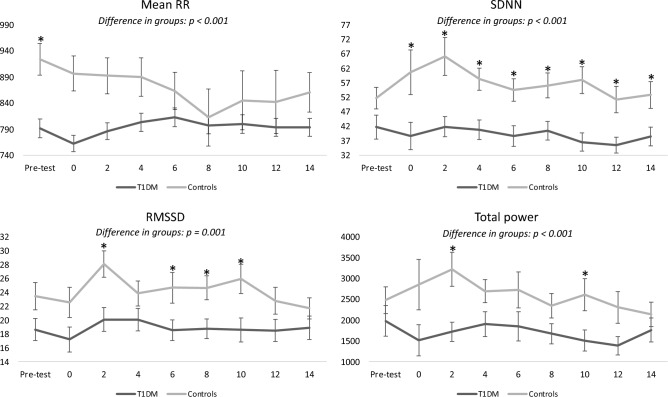
Dynamic HRV responses to cold tonic pain. Changes in HRV measures over time for the T1DM group (dark line) and controls group (grey line). Each time point represents a 2-min interval except the pre-test, which represents a 4-min epoch. Timepoint 0 represents the 2-min tonic cold pain exposure epoch. The p-values for unadjusted differences in groups (curves) are included. Data presented as raw data and as mean ± standard error. Significance levels at fixed time points are according to data presented in Table 3. *SDNN* Standard deviation of NN intervals, *RMSSD* root mean square of successive differences in NN intervals.

Exposure to tonic cold pain revealed dynamic differences between healthy controls and individuals with T1DM and polyneuropathy before, during, and after exposure to tonic cold pain, in particular in SDNN (p < 0.001), RMSSD (p = 0.012), total power (p < 0.001), LF/HF (p = 0.041), and mean RR (p < 0.001), see Fig. 1. We controlled for age and BMI to avoid confounding, which resulted in an unsignificant level for LF/HF. Detailed comparisons between the initial, sustained, and recovery dynamic changes to tonic cold pain exposure are displayed in Table 3.

**Table 3:** Dynamic HRV responses.

Dynamic HRV responses			Pre-test	Initial response		Sustained response			Recovery			p-value**
			− 4 to 0 min	0 to 2 min	2 to 4 min	4 to 6 min	6 to 8 min	8 to 10 min	10 to 12 min	12 to 14 min	14 to 16 min	
Crude differences	Mean RR (ms)	p value (ΔΔ)	**0.000**	0.058	0.079	0.357	0.646	0.728	0.529	0.401	0.064	**< 0.001**
		CI	60.2; 195.5	− 2.6; 154.1	− 8.3; 150.2	− 44.4; 123.1	− 65.3; 105.2	− 83.0; 118.9	− 67.7; 131.8	− 58.7; 146.8	− 4.0; 142.3	
	SDNN (ms)	p value (ΔΔ)	0.073	**0.023**	**0.002**	**0.003**	**0.005**	**0.007**	**0.000**	**0.003**	**0.010**	**< 0.001**
		CI	− 0.9; 20.6	2.9; 37.6	7.7; 35.5	5.0; 25.2	4.4; 25.5	4.0; 24.8	10.3; 32.4	5.5; 26.4	3.5; 25.7	
	RMSSD (ms)	p value (ΔΔ)	0.073	0.053	**0.002**	0.147	**0.027**	**0.024**	**0.018**	0.093	0.202	**0.001**
		CI	− 0.4; 9.1	− 0.1; 11.0	2.8; 12.9	− 1.2; 8.3	0.7; 11.4	0.7; 10.4	1.0; 11.2	− 0.7; 9.4	− 1.7; 8.1	
	Total power (ms^2^)	p value (ΔΔ)	0.294	0.101	**0.006**	0.217	0.161	0.161	**0.025**	0.056	0.352	**< 0.001**
		CI	421.3; 1391.9	− 225.6; 2548.9	351.6; 2121.7	− 296.2; 1302.3	− 301.2; 1813.1	− 219.7; 1323.9	128.1; 1887.7	− 20.6; 1668.0	− 391.4; 1098.7	
	VLF (ms^2^)	p value (ΔΔ)	0.299	0.225	0.158	0.432	0.366	0.172	0.060	0.044	0.299	0.130
		CI	− 297.4; 967.2	− 454.1; 1932.9	− 179.6; 1104.3	− 350.1; 817.9	− 449.6; 1219.8	− 324.7; 1821.7	− 31.1; 1581.0	15.3; 1162.5	− 224.5; 730.1	
	LF (ms^2^)	p value (ΔΔ)	0.326	0.075	**0.044**	0.246	0.241	0.299	0.382	0.310	0.578	0.671
		CI	− 164.6; 495.7	− 44.6; 932.2	16.0; 1196.9	− 198.7; 775.3	− 194.2; 773.8	− 169.4; 552.0	− 222.2; 580.2	− 202.8; 638.2	− 319.6; 573.0	
	HF (ms^2^)	p value (ΔΔ)	0.671	0.146	0.368	0.752	0.650	0.446	0.758	0.734	0.879	0.133
		CI	− 85.8; 55.3	− 44.5; 299.6	− 45.6; 123.2	− 105.2; 75.9	− 72.1; 115.6	− 50.1; 114.0	− 89.3; 122.6	− 58.1; 82.5	− 74.5; 63.7	
	LF/HF (ms^2^)	p value (ΔΔ)	**0.020**	0.051	**0.042**	**0.006**	0.188	0.396	0.334	0.294	0.931	**0.041**
		CI	0.7; 7.6	− 0.0; 10.0	0.2; 8.7	1.6; 9.3	− 3.3; 16.6	− 1.6; 4.0	− 2.2; 6.4	− 1.6; 5.3	− 5.5; 5.0	
Adjusted differences*	Mean RR (ms)	p value (ΔΔ)	**0.000**	0.106	0.165	0.572	0.953	0.992	0.754	0.601	0.141	**< 0.001**
		CI	50.5; 170.5	− 12.4; 128.2	− 21.9; 128.6	− 53.9; 97.5	− 75.5; 80.2	− 93.6; 94.5	− 78.2; 108.0	− 73.3; 126.6	− 17.1; 120.2	
	SDNN (ms)	p value (ΔΔ)	0.073	**0.023**	**0.004**	**0.005**	**0.010**	**0.010**	**0.000**	**0.002**	**0.016**	**0.003**
		CI	−.9; 19.3	2.6; 36.1	6.8; 34.7	4.4; 24.1	3.3; 25.1	3.2; 23.6	9.4; 31.7	5.5; 24.7	2.6; 25.0	
	RMSSD (ms)	p value (ΔΔ)	0.185	0.120	**0.011**	0.332	0.062	0.082	**0.048**	0.183	0.399	**0.002**
		CI	− 1.6; 8.5	− 1.2; 10.1	1.5; 12.2	− 2.6; 7.7	−.2; 10.4	−.6; 9.7	.0; 10.3	− 1.6; 8.3	− 2.9; 7.4	
	Total power (ms^2^)	p value (ΔΔ)	0.350	0.124	**0.016**	0.256	0.203	0.229	**0.040**	0.069	0.469	**0.014**
		CI	− 483.0; 1362.3	− 302.5; 2501.2	222.6; 2127.6	− 320.1; 1204.3	− 380.9; 1790.3	− 300.9; 1258.6	43.9; 1859.3	− 59.7; 1587.6	− 508.5; 1103.8	
	VLF (ms^2^)	p value (ΔΔ)	0.340	0.254	0.191	0.515	0.405	0.209	0.060	0.061	0.387	0.158
		CI	− 333.5; 966.9	− 505.9; 1912.4	− 212.5; 1064.8	− 398.7; 795.0	− 485.6; 1204.3	− 395.1; 1805.9	− 31.6; 1521.7	− 26.5; 1138.1	− 284.9; 735.2	
	LF (ms^2^)	p value (ΔΔ)	0.411	0.087	0.064	0.282	0.234	0.410	0.472	0.399	0.642	0.656
		CI	− 215.2; 525.9	− 62.6; 915.9	− 34.4; 1213.2	− 223.2; 766.2	− 179.5; 735.2	− 230.1; 563.2	− 282.0; 608.4	− 265.2; 666.5	− 358.2; 581.4	
	HF (ms^2^)	p value (ΔΔ)	0.544	0.205	0.488	0.622	0.753	0.600	0.864	0.912	0.707	0.129
		CI	− 92.2; 48.6	− 64.9; 302.6	− 55.3; 115.8	− 115.0; 68.8	− 75.5; 104.4	− 61.3; 106.1	− 95.5; 113.7	− 69.2; 77.4	− 81.1; 55.0	
	LF/HF (ms^2^)	p value (ΔΔ)	**0.007**	**0.039**	**0.027**	**0.005**	0.136	0.156	0.294	0.190	0.913	0.057
		CI	1.3; 8.0	0.3; 11.3	0.6; 9.7	1.9; 10.5	− 2.3; 17.1	− 0.7; 4.5	− 2.3; 7.5	− 1.2; 6.0	− 4.7; 5.2	

Detailed values of differences in HRV measures, enabling comparison between T1DM and healthy controls group (ΔΔ) for pre-test and each consecutive 2-min epoch. 0–2 min represents the tonic cold pain stimulation epoch. Table based on raw data supplied with model-based p-values using bootstrapping with 1000 replications, with* and without adjusting for age and BMI.

*CI* confidence interval.

**p-value for difference in groups (curves).

Significant values are in bold.

Individuals with T1DM and polyneuropathy had an adynamic response compared to healthy controls, lacking the flexible, adaptable initial/immediate response to tonic cold pain. When comparing differences between groups, the following dynamic responses were lower in initial response phase in the T1DM group: SDNN (0% vs. 28% increase, p = 0.002), RMSSD (4% vs. 20% increase, p = 0.002), total power (13% decrease vs. 30%, p = 0.006), LF (11% vs. 73% increase, p = 0.044), contrasting that the LF/HF response was higher in the T1DM group (15% vs. 6% increase).

## Discussion

In line with existing literature^29,30^, we found that HRV time- and frequency domain measures derived from 24-h ECG significantly discriminated our cohorts of individuals with T1DM and polyneuropathy and age- and sex-matched healthy controls, indicating diabetes-induced dysautonomia. We further investigated the neurocardiac adaptability by investigating the electrocardiographically dynamic responses representing HRV measures before, during, and after exposure to tonic cold pain. Compared to healthy, we found convincingly adynamic responses in the T1DM group, evident as a delayed increase in SDNN and decreased parasympathetic regulation, contributing to a more chronometric heart rhythm. The findings reveal impaired neurocardiac adaptability to rapidly adjust to potential threats (here mimicked with cold pain exposure), causing physical or psychological alterations, and consequently, maintained homeostasis is mistrusted.

### Baseline characteristics

This study included 21 healthy and 48 individuals with T1DM and confirmed DSPN (decreased nerve conduction velocities and thermal pain tolerance), increased heart rate and systolic blood pressure, and 90% of had orthostatic hypotension, indicating both peripheral and severe autonomic nerve dysfunction. This was further supported by the mean clinical risk score QRISK3 of 20%, indicating a relatively high risk of cardiovascular events within the next 10 years. All participants in the T1DM group had diabetic retinopathy, and the number of participants with a UACR above > 30 mg/g (lower level of microalbuminuria) was larger in the T1DM group, reflecting the systemic presence of microvascular complications. Antihyperlipidemic and antihypertensive treatment is recommended in the diabetes treatment guidelines. This explains why a larger percentage of participants received these treatments in the T1DM group, reflected as lower levels of LDL than healthy.

### Autonomic regulation reflected in HRV

The HRV measures are an emergent property assessing the neurocardiac adaptability, reflecting the heart-brainstem interactions and regulated through the non-linear regulated autonomic nervous system^10^. HRV describes the fluctuations in the time intervals between consecutive heartbeats. Mean RR is simply the time of the inter-beat interval. SDNN is the standard deviation of the RR-intervals and is considered the most robust parameter to quantify the inter-beat variability and neurocardiac adaptability, and it reflects both sympathetic and parasympathetic modulation in response to physiological influences^38^. RMSSD and HF are strongly believed to represent central parasympathetic regulation^8^, in contrast to the LF component of HRV, which reflects both sympathetic and parasympathetic activity. However, the utility of the LF component of HRV is largely debated^39–41^. The LF/HF ratio is believed to reflect the sympathovagal balance or sympathetic modulations^8^. According to Shaffer et al., a low LF/HF reflects parasympathetic dominance, whereas a high LF/HF reflects sympathetic dominance^10^. Finally, the indices have proven valuable in assessing individual risk stratification for cardiovascular events and the degree of neurocardiac dysautonomia^6,42^.

Our dynamic assessments provided the possibility to interpret the cold pain-induced comprehensive autonomic response, comparable to the assessment of orthostatic hemodynamics in response to, e.g., standing or tilting, representing initial, sustained, and recovery phases^43^. Compared to healthy, the diabetes group experienced less unpleasantness, but there was no difference in pain response to tonic cold pain or thermal heat stimulation. This rules out that peripheral neuropathies modulated the afferent upstream activation of the tonic pain experience, thereby biasing our HRV data. Consequently, as both groups were exposed to equipotent peripheral pain experiences, our findings of decreased parasympathetic response to tonic cold pain in the diabetic group are supported by impaired neurocardiac regulation, evident as a delayed increase in SDNN. Taken together, the adynamic HRV responses in T1DM to tonic pain indicate central dysautonomia, even when controlling for age and BMI.

### Characteristics of HRV (ultra-short vs. long-term recordings)

We used ultra short-term HRV epochs (2 min) to assess the dynamic neurocardiac regulation. Such short epochs have less variability compared to 24-h recordings. Even though they are influenced by sinus arrhythmia, the baroreceptor reflex (negative feedback control of blood pressure), and rhythmic changes in vascular tone^8,37^, the 24-h recordings are further influenced by circadian rhythms, body core temperature, metabolism, sleep pattern, and the regulation of the renin-angiotensin system^8,37^, thereby providing the highest possible variability. The 24-h recordings are the gold standard for clinical HRV assessments because they are more accurate in predicting cardiovascular events than short recordings. Even though we use identical mathematical formulas to assess HRV from 24-h and short-term recordings, they represent complementary physiological measures. Simplistically, RMSSD and HF have been suggested to quantify the portion of parasympathetic regulation and used to estimate the sympathovagal balance^8^. Such indices are often used to assess individual risk stratification for cardiovascular events and the degree of neurocardiac dysautonomia^42^.

An example is the LF/HF ratio, which may describe the sympathovagal balance under controlled conditions^44^. However, the measure should be interpreted cautiously in situations where the neurocardiac regulation is altered in response to external situations, such as the cold pressor test, primarily because the total power (sum of the energy in VLF, LF, and HF bands) is highly variable and changes in response to external stimuli that challenges homeostasis^10^. In that perspective, the short-term data reflect vulnerability to underreport differences. Nonetheless, the recordings were analyzed as a series of 2-min epochs and revealed significant differences providing robust and complementary information on the neurocardiac adaptability to cold pain exposure.

### Healthy controls vs. T1DM in 24-h HRV recordings

The 24-h recordings revealed impaired neurocardiac regulation, and the shown diminished RMSSD and HF support the diagnosis of cardiovascular autonomic neuropathy (CAN) characterized by severe sympathetic dominance and parasympathetic withdrawal. Even though HRV measures are known to be reduced in individuals with T1DM in comparison to healthy controls^9,28–31,45^, conflicting results exist, which may indicate co-activation or co-inhibition of the two tonically activated branches, allowing rapid physiological neurocardiac adaptation in response to the surroundings. Furthermore, attenuated HRV responses at rest, but not in response to cold pain, were shown in a cohort with 20 participants with type 2 diabetes compared to 10 healthy controls^33^. Consequently, LF/HF may not always index autonomic balance accurately^7,10,32,33^.

To our surprise, total power, VLF, LF, and HF components of the 24-h HRV measures were correlated to the mean QRISK3, supporting the relevance of assessing neurocardiac regulation with HRV measures. Interestingly, based on the ultra-short epochs, QRISK3 scores negatively correlated with SDNN, total power, VLF, LF, and HF between 2 and 6 min post-exposure, underlining that parasympathetic withdrawal and adynamic appearance of other HRV measures are associated with increased risk of having a cardiovascular event.

### Complementary neurocardiac measures

The arterial baroreceptors buffer acute fluctuations in blood pressure during e.g., sympathetic dominance, deep breathing, or postural changes by transducing increased vascular distension into nervous electrical activity, which actuates parasympathetic activation and sympathetic inhibition^44^. Baroreceptor activity is activated through high-pressure (aortic arch) or both low- and high-pressure (carotid sinus) baroreceptors, triggering distinct pathways, the so-called cardio-vagal, the cardio-sympathetic or the vaso-sympathetic pathway^46^. We showed decreased CSB in T1DM, constituting the afferent branch of the cardiac-brainstem communication. Interestingly, we only found a tendency towards decreased CVT, constituting the efferent branch of the cardiac-brainstem communication, but previously it has been reported that CVT is reduced in T1DM^34^. Both findings are in accordance with the literature, plausibly explained by damage to central or peripheral (afferent and/or efferent) parts of the cardiac-brainstem circuit^47^.

### Exposure to tonic cold pain

We found no group differences between healthy and T1DM in perceived pain, and hence, believe that the individuals were exposed to an equipotent painful stimulus, with a proportional autonomic response. Exposure to cold tonic pain is perceived with high inter-individual variability, which has classically been considered to activate the sympathetic response because it is supported by elevated adrenaline and noradrenaline levels, increased heart rate or blood pressure^13–15,17–24^. Nevertheless, novel data reveal that approximately 30% of healthy subjects respond to cold pain exposure with parasympathetic dominance^48^, suggesting a more complex response, where an individual physiological meaningful linear, and non-linear co-activation of the sympathetic and parasympathetic system is present.

### HRV responses in healthy following to exposure to cold pain

We refined the existing methodologies and measured mean HRV responses in ultra-short epochs (2 min) before, during, and up to 14 min after cold tonic pain (2 min at 2 °C) to allow a more dynamic interpretation of the neurocardiac regulation in response to cold pain exposure. The experimental setting is comparable to, e.g., provocative tests such as treadmill tests, in which stressing the neurocardiac central regulatory system can reveal changes that otherwise may not be visible under resting conditions. In the healthy cohort, we show convincingly dynamic physiological adaptive capacity of the heart, evidenced by increased SDNN and RMSSD, supported by Sanchez-Gonzalez et al., who also found increased RMSSD in response to cold pain^49^. In contrast, Jarczewski et al. found a decrease in RMSSD^14^, and MacArtney et al. did not show changes in SDNN or RMSSD in response to the cold pressor test^16^. Differences in used methodologies and duration of the analyzed ECG epochs could explain these discrepancies. Example, Jarczewski et al. measured the mean HRV responses in a 10-min epoch following a similar cold pressor test (2 min at 0–4 °C), whereas MacArtney et al. measured mean HRV responses in a 5-min epoch during the cold pressor test (5 min, 5 °C)^16,17^. Furthermore, since especially parasympathetic modulation decreases with increasing age^50^, and as our cohort is approximately double the age as those in the Jarczewski et al. and MacArtney et al., the need for matched controls to reliably compare the HRV responses between our two groups is emphasized^16,17^.

The initial group level response to tonic cold pain showed an increase in total power and LF/HF ratio, indicating more LF (or less HF) power, plausibly revealing increased sympathetic response in response to cold pain exposure. The literature shows ambiguous results, some report increased LF^25,49^ and HF power^25,49,51^ within the first 2 min after hand immersion into ice water, whereas others describe decreased LF^17^ and HF^52^, which may be explained by individual responses, different methodologies, different central brainstem regulations (co-inhibition or reciprocal activation of the two branches), sympathetic ceiling, withdrawal, or enhanced compensatory parasympathetic tone.

### HRV responses in T1DM with polyneuropathy following exposure to cold pain

Throughout the entire recording in T1DM, SDNN did not differ from the pre-test epoch indicating chronometrical heart rhythm, regardless of tonic cold pain exposure. This is supported by decreased mean RR and RMSSD in the initial response to cold pain exposure, indicating rapid sympathetic dominance, which interestingly is characterized by decreased total power, LF and LF/HF ratio decreased, and unchanged HF. At first sight, one could speculate that it solely represents sympathetic dominance, but it could also indicate that parasympathetic tone is at a minimum, and that is why it cannot be modulated more—even by a provocative test. Taken together, it indicates altered central neurocardiac regulation and the presence of cardiovascular autonomic neuropathy^3,6^. As a late sustained response in the recovery phase, sympathetic withdrawal or relatively enhanced parasympathetic activity was shown by increased RR intervals, but most evident is the presence of adynamic responses in SDNN, RMSSD, and total power, indicating non-adaptability of the neurocardiac regulation. To our knowledge, no previous studies have investigated such dynamic HRV response to tonic cold pain exposure in T1DM, unmasking the complexity of the concomitant sympathetic and parasympathetic autonomic regulation.

### Differences in HRV responses between groups

In comparison to healthy, we found that time-domain (SDNN and RMSSD) and frequency-domain (total power, LF) responses to cold pain exposure were unphysiologically adynamic, leading to decreased cardiac adaptability, chronometrical HR, and ultimately diminished ability to counteract or adapt to threatening external factors. Interestingly, in response to tonic cold pain exposure, both T1DM and healthy controls experienced a rapid decrease in the LF/HF ratio; however, total power was only diminished in the T1DM group. As the parasympathetic response was unchanged, this finding indicates differences in sympathetic responses, which were less powerful and less adaptable in the T1DM group. This is further supported by a cross-sectional study conducted in a cohort of 52 participants with diabetes and 15 years of diabetes duration, where no alterations in HR and blood pressure were shown in response to cold pain exposure^7^. Taken together both branches show impaired responses. The parasympathetic response is adynamic and unadaptable, but the sympathetic response can still, but to a lesser degree than normal, be modulated.

### Strengths and limitations

This is the first time that the dynamic HRV response to tonic cold pain has been investigated in a cohort of individuals with T1DM and polyneuropathy and compared with an age- and sex-matched cohort of healthy controls. Unsurprisingly, we showed convincing differences in the HRV measures based on 24-h recordings, supporting dysautonomia in the T1DM group. Furthermore, we showed robust group differences in the capacity of adapting the neurocardiac regulation in responses to tonic cold pain exposure. However, the first obvious limitation is that the participants with T1DM have long-term diabetes and severe verified polyneuropathy and the majority have orthostatic hypotension, indicative of severe CAN. Thus, our findings support clinical findings, but unfortunately, cardiovascular autonomic reflex tests were not carried out to diagnose cardiovascular autonomic neuropathy, hampering the ability to stratify the cohort according to the CAN stage. Secondly, 2 min epochs are ultra-short, making them vulnerable to physiological noise such as sinus arrhythmia, respiration patterns, coughing, certain movements, or stress response caused by the cold pressor test. However, data was consistently calculated within meaningful predefined intervals, and HRV analyses were conducted by the same person by use of validated software, minimizing the inter- and intra-observer differences. Thirdly, including the Poincaré plot could have revealed non-linear dynamics of consecutive RR intervals and thus provided additional information on the heart rate variability changes, but this was not included in the study. Fourthly, it has been shown in healthy that the cold pressor test causes high inter-individual variability, reflected in primarily parasympathetic and sympathetic responses, and thus, group comparisons yield a source of error. Nevertheless, in comparison to healthy, we showed that the parasympathetic response in T1DM was convincingly adynamic, and the magnitude of the adaptability of the sympathetic response was decreased. Lastly, it could have strengthened the interpretation of the neurocardiac regulation if novel parameters of sympathetic and parasympathetic activation, such as periodic dynamic depolarization of the T-wave and deceleration capacity, were used, but these were not available in this study.

## Conclusion

In T1DM, an attenuated sympathovagal response was shown as convincingly adynamic parasympathetic responses and diminished sympathetic adaptability, causing chronometric heart rhythm and rigid neurocardiac regulation threatening homeostasis. The findings associate with an increased risk of cardiovascular disease, emphasizing the clinical relevance. The method quantifies and illustrates the complex autonomic regulation where physiological meaningful linear and non-linear co-activation may unmask dysautonomia, ultimately in earlier and silent stages.

## Data Availability

The data used during the current study are not publicly available but are available from the corresponding author on reasonable request.

## Abbreviations

CAN: Cardiac autonomic neuropathy
CSB: Cardiac sensitivity to the baroreflex
CVT: Cardiac vagal tone
DBS: Diastolic blood pressure
DPSN: Distal symmetrical polyneuropathy
HF: High-frequency
HR: Heart rate
HRV: Heart rate variability
LF: Low-frequency
LF/HF: Low-frequency/high-frequency ratio
RMSSD: Root mean square of successive differences in NN intervals
SBS: Systolic blood pressure
SDANN: Standard deviation of all NN intervals for each 5-min segment of a 24-h recording
SDNN: Standard deviation of NN intervals
SDNNI: Mean of the standard deviation of all NN intervals for each 5-min segment of a 24-h recording
T1DM: Type 1 diabetes
VLF: Very-low-frequency
